# Multitemporal lidar captures heterogeneity in fuel loads and consumption on the Kaibab Plateau

**DOI:** 10.1186/s42408-022-00142-7

**Published:** 2022-08-09

**Authors:** Benjamin C. Bright, Andrew T. Hudak, T. Ryan McCarley, Alexander Spannuth, Nuria Sánchez-López, Roger D. Ottmar, Amber J. Soja

**Affiliations:** 1 Rocky Mountain Research Station, USDA Forest Service, 1221 S Main Street, Moscow, ID 83843 USA; 2 College of Natural Resources, University of Idaho, 875 Perimeter Drive, Moscow, ID 83844 USA; 3 Kaibab National Forest, USDA Forest Service, 800 S 6th Street, Williams, AZ 86046 USA; 4 Pacific Northwest Research Station, USDA Forest Service, 400 N 34th Street, Suite 201, Seattle, WA 98103 USA; 5 National Institute of Aerospace, 100 Exploration Way, Hampton, VA 23666 USA; 6 NASA Langley Research Center, 21 Langley Blvd MS 420, Hampton, VA 23681 USA

**Keywords:** Forest fuels, Canopy fuels, Surface fuels, Fuel mapping, Fuel consumption, Post-fire fuel dynamics, Remote sensing, Airborne lidar

## Abstract

**Background:**

Characterization of physical fuel distributions across heterogeneous landscapes is needed to understand fire behavior, account for smoke emissions, and manage for ecosystem resilience. Remote sensing measurements at various scales inform fuel maps for improved fire and smoke models. Airborne lidar that directly senses variation in vegetation height and density has proven to be especially useful for landscape-scale fuel load and consumption mapping. Here we predicted field-observed fuel loads from airborne lidar and Landsat-derived fire history metrics with random forest (RF) modeling. RF models were then applied across multiple lidar acquisitions (years 2012, 2019, 2020) to create fuel maps across our study area on the Kaibab Plateau in northern Arizona, USA. We estimated consumption across the 2019 Castle and Ikes Fires by subtracting 2020 fuel load maps from 2019 fuel load maps and examined the relationship between mapped surface fuels and years since fire, as recorded in the Monitoring Trends in Burn Severity (MTBS) database.

**Results:**

*R*-squared correlations between predicted and ground-observed fuels were 50, 39, 59, and 48% for available canopy fuel, 1- to 1000-h fuels, litter and duff, and total surface fuel (sum of 1- to 1000-h, litter and duff fuels), respectively. Lidar metrics describing overstory distribution and density, understory density, Landsat fire history metrics, and elevation were important predictors. Mapped surface fuel loads were positively and nonlinearly related to time since fire, with asymptotes to stable fuel loads at 10–15 years post fire. Surface fuel consumption averaged 16.1 and 14.0 Mg ha^− 1^ for the Castle and Ikes Fires, respectively, and was positively correlated with the differenced Normalized Burn Ratio (dNBR). We estimated surface fuel consumption to be 125.3 ± 54.6 Gg for the Castle Fire and 27.6 ± 12.0 Gg for the portion of the Ikes Fire (42%) where pre- and post-fire airborne lidar were available.

**Conclusions:**

We demonstrated and reinforced that canopy and surface fuels can be predicted and mapped with moderate accuracy using airborne lidar data. Landsat-derived fire history helped account for spatial and temporal variation in surface fuel loads and allowed us to describe temporal trends in surface fuel loads. Our fuel load and consumption maps and methods have utility for land managers and researchers who need landscape-wide estimates of fuel loads and emissions. Fuel load maps based on active remote sensing can be used to inform fuel management decisions and assess fuel structure goals, thereby promoting ecosystem resilience. Multitemporal lidar-based consumption estimates can inform emissions estimates and provide independent validation of conventional fire emission inventories. Our methods also provide a remote sensing framework that could be applied in other areas where airborne lidar is available for quantifying relationships between fuels and time since fire across landscapes.

## Resumen

**Antecedentes:**

La caracterización de la distribución física de los combustibles a través de paisajes heterogéneos es necesaria para entender el comportamiento del fuego, contabilizar las emisiones de humo, y manejar los ecosistemas para su resiliencia. Las mediciones mediante sensores remotos a varias escalas, aportan mapas para mejorar modelos de fuegos y dispersión de humos. Las mediciones con LIDAR aerotransportados que determinan directamente variaciones en altura y densidad de la vegetación, han probado ser especialmente útiles para el mapeo de la carga y el consumo de combustible a escala de paisaje. Predijimos la carga de combustibles en la planicie de Kaibab en el norte de Arizona, en los EEUU, estimamos el consumo a través de los incendios de Castle e Ikes de 2019, mediante la substracción de la carga de mapas de carga del 2020 menos los de 2019, y examinamos las relaciones entre el mapeo de los combustibles superficiales y años desde el fuego, registrados en la base de datos titulada Monitoreo de las Tendencias de la Severidad de los incendios (MTBS).

**Resultados:**

Las correlaciones de R^2^ entre valores de cargas predichos y aquellos de observaciones de campo fueron 50, 39, 59, y 48% para combustible disponible en el dosel, combustibles de 1 a 1000 h, mantillo y hojarasca por debajo del mantillo (duff), y combustible total superficial (la suma de combustibles de 1 a 1000 h y del mantillo y la hojarasca subyacente), respectivamente. Las medidas del LIDAR que describían la distribución del dosel y su densidad, la densidad del sotobosque, las medidas históricas de fuego provistas por el Landsat y la altura (elevación) fueron predictores importantes. Las cargas de combustibles mapeadas fueron positivamente y no linealmente relacionadas al tiempo desde el fuego, con asíntotas hacia cargas de combustible estables entre 10 y 15 años post fuego. El consumo de la carga de combustibles en superficie promedió 16,1 y 14,0 Mg por ha para los incendios de Castle e Ikes, respectivamente y fue positivamente correlacionada con la diferencia normalizada de la relación de quema (dNBR). Estimamos que el consumo del combustible superficial fue de 125,3 ± 54,6 Gg para el incendio de Castle y 27,6 ± 12,0 Gg para la porción del incendio de Ikes (42%), del cual los datos de LIDAR aerotransportados (pre y post fuego), estaban disponibles.

**Conclusiones:**

Demostramos y reforzamos que tanto el dosel como los combustibles superficiales pueden ser predichos y mapeados con una moderada precisión usando datos de LIDAR aerotransportados. Las medidas históricas de fuego provistas por el Landsat ayudaron a determinar la variación espacial y temporal de la carga de los combustibles superficiales y nos permitieron describir tendencias temporales en las cargas de combustible superficiales. Nuestros mapas y métodos de consumo y cargas de combustible son de utilidad para los gestores de recursos e investigadores que necesitan de estimaciones amplias de carga de combustible y emisiones a escala de paisaje. Los mapas de carga de combustibles basados en sensores remotos activos pueden ser usados para informar sobre decisiones de manejo de combustible y determinar metas de estructuras de cargas, promoviendo de esa manera la resiliencia del ecosistema. Las estimaciones de consumo basadas en LIDAR multitemporal pueden informar sobre estimaciones de emisiones y proveer de una validación de inventarios convencionales de emisiones por fuegos. Nuestros métodos también proveen de un marco conceptual de sensores remotos que pueden ser aplicados en otras áreas donde el LIDAR aerotransportado está disponible para cuantificar relaciones entre combustibles y tiempo desde el fuego en diferentes paisajes.

## Background

Land managers and researchers require fuel load measurements to manage fuels for ecosystem resilience (Covington et al. 1994; Graham et al. 2004), predict fire behavior (Countryman 1972; Alexander and Cruz 2013; Keane 2015), and quantify fire emissions (Seiler and Crutzen 1980; Leenhouts 1998; French et al. 2004). Remote sensing data can facilitate spatially explicit estimates of fuel loads that would be difficult to obtain from inherently high heterogeneity fuel distributions that are impractical to characterize from in situ measurements alone (Keane et al. 2001; Arroyo et al. 2008; Keane 2015). Integrated modeling methodologies often involve relating in situ measurements of fuel loads, such as tallies along transects (Brown 1974) or destructive samples (Hawley et al. 2018), to remotely sensed data and other ancillary data sources that provide synoptic coverage (Keane et al. 2001). For many applications, such as landscape-level management of fuels, fuel load estimates at coarser spatial scales (20 to 30 m) are sufficient and perhaps preferred (e.g., Rollins 2009; Reeves et al. 2009). For some recent fire science investigations using physically based models, more highly resolved, three-dimensional representations of fuel loads are needed to better model and understand fire behavior (Hiers et al. 2009, 2020; Rowell et al. 2016). Because fuels are dynamic in space and time, especially following disturbance, methods that quantify the relationship between fuel loads and time since disturbance are needed to monitor and model fuel accumulation across landscapes. Methods modeling the dynamic relationships between disturbance and fuel loading over space and time can alleviate the need for annual fuel loading monitoring efforts. Such methods can also help wildland fire managers assess potential fire behavior with geospatial fire history information which is common in many national forests around the United States. Mapping and assessing the heterogeneous fuel loading trajectories across a given landscape can help planning efforts aimed at preventing undesirable impacts to human and natural communities.

Light detection and ranging (lidar) actively measures live and dead vegetation structure and is therefore particularly well-suited, relative to passive optical sensors, for quantifying forest fuel loads at various spatial scales and height strata (Arroyo et al. 2008); common fuel strata definitions include canopy, shrub, herbaceous, downed woody, litter, and duff layers (Ottmar et al. 2007). Terrestrial lidar, in which the lidar sensor is mounted on or near ground level, has been used to estimate canopy, shrub, and herbaceous fuel loads at fine spatial scales (spatial resolutions of 1 m or less, e.g., Loudermilk et al. 2009; Skowronski et al. 2011; Hudak et al. 2020, Rowell et al. 2020). To generate coarser-scale fuel load maps (spatial resolutions of 20 m or greater) of various fuel strata across landscapes, airborne lidar is commonly used (e.g., Andersen et al. 2005; Erdody and Moskal 2010; Hermosilla et al. 2013; Hudak et al. 2015, 2016). Spaceborne lidar has also been applied to mapping of various fuel strata (e.g., García et al. 2012; Peterson et al. 2013; Leite et al. 2022). When pre- and post-fire lidar data are available, fuel load estimates can be differenced to estimate consumption (McCarley et al. 2020; Hudak et al. 2020), which can be useful for investigating fire effects and fuel-fire-emissions relationships (Hudak et al. 2015; McCarley et al. 2020).

Previous studies using airborne and spaceborne lidar for fuel load estimation have most often predicted canopy fuel loads. Subcanopy (shrub, herbaceous, downed woody, litter, and duff) fuel loads have been predicted less often and less accurately (Seielstad and Queen 2003; Pesonen et al. 2008; Jakubowski et al. 2013; Hudak et al. 2015, 2016; Price and Gordon 2016; Bright et al. 2017; Stefanidou et al. 2020; McCarley et al. 2020; Mauro et al. 2021; Alonso-Rego et al. 2021; Leite et al. 2022). Limitations to measuring subcanopy fuels include (1) occlusion and attenuation by the overstory so that near-ground fuel structure is sampled unreliably, (2) insufficient horizontal point density and/or vertical accuracy (often ~ 15 cm) to quantify near-surface fuel heights, (3) inability to directly measure litter and duff depth, and (4) the often intrinsic, high heterogeneity of subcanopy fuels across space that makes reliable in situ sampling and therefore modeling difficult (Keane et al. 2001; Keane 2015). Despite these challenges, previous studies have reported useful prediction accuracies and have concluded that subcanopy fuel load estimates derived from airborne and spaceborne lidar have utility for managers.

Here we predicted and mapped available canopy fuel (foliage weight plus 50% of small branch weight) and surface fuels (downed woody, litter, and duff) across a landscape in northern Arizona, USA, using in situ field observations, multitemporal airborne lidar, and Landsat-derived fire history metrics (number of past fires (NPF) and years since fire (YSF)). By differencing pre- and post-fire fuel load maps, we estimated fuel consumption for two fires, the Castle and Ikes Fires of 2019. Few previous studies have estimated fuel consumption across a landscape with multitemporal airborne lidar (e.g., Wang and Glenn 2009; Alonzo et al. 2017; Hoe et al. 2018; Hu et al. 2019; Skowronski et al. 2020; McCarley et al. 2020). We also examine the relationship between lidar-derived surface fuel load maps and fire history and present a remote sensing framework for quantifying temporal dynamics in surface fuel accumulation, which, to our knowledge, no previous study has done.

## Methods

### Study area

**Fig. 1:**
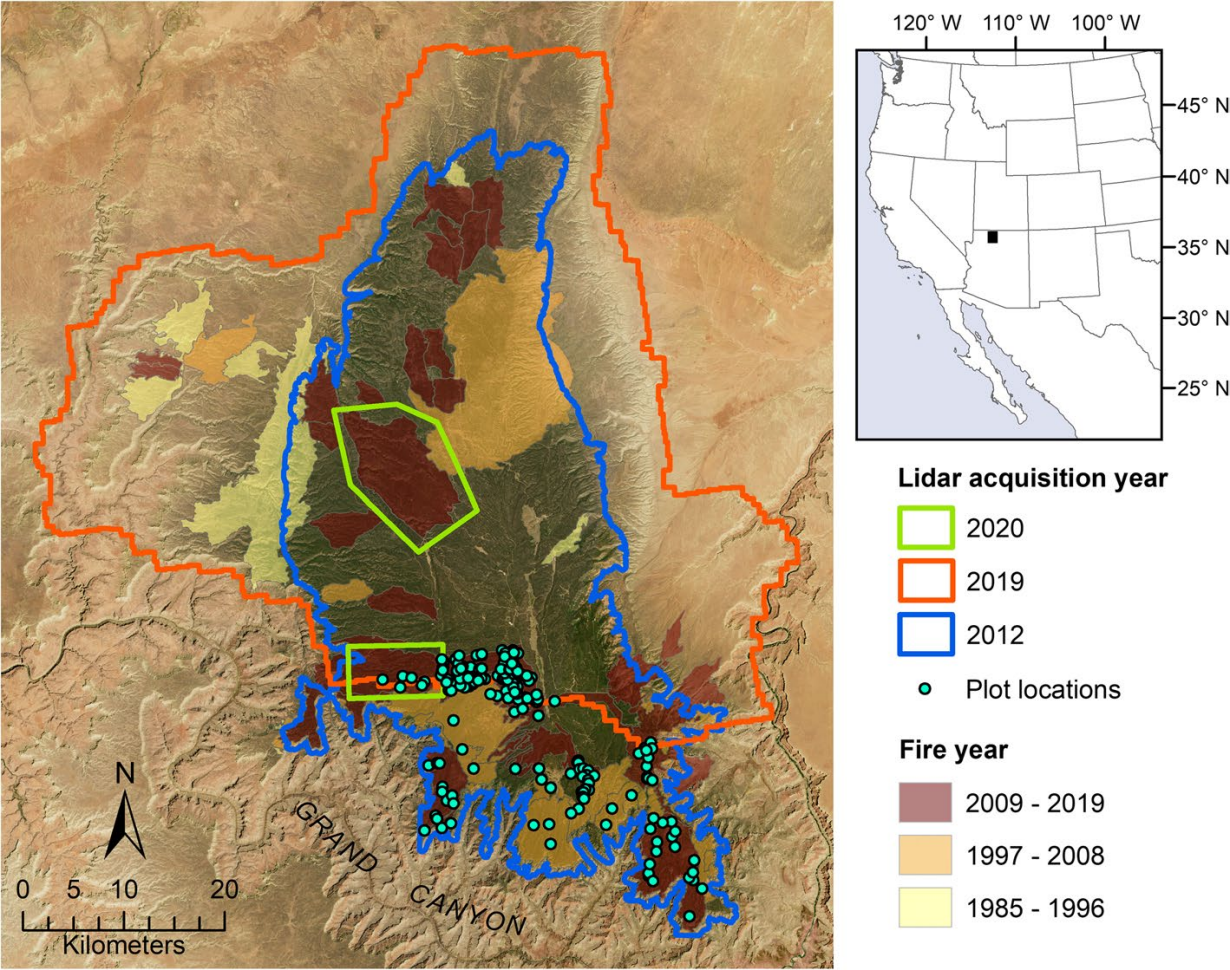
The Kaibab Plateau in northern Arizona overlaid with airborne lidar extents and past fire perimeters from the Monitoring Trends in Burn Severity (MTBS) database. The 2020 lidar was two parcels acquired across the entire 2019 Castle Fire (within the northern 2020 lidar polygon) and a portion of the 2019 Ikes Fire (within the southern 2020 lidar rectangle). True-color background imagery is from Landsat 8

Our study area spanned the Kaibab Plateau on the north rim of the Grand Canyon in northern Arizona, USA (Fig. 1), which is administered by the United States National Park Service (USNPS) and United States Forest Service (USFS). Annual precipitation increases with elevation to dictate dominant vegetation type across the plateau, with shrublands occurring at lower elevations (minimum of 860 m in our study area), woodlands occurring at intermediate elevations, and forests occurring at higher elevations (maximum of 2800 m in our study area). Forest types across the plateau, in order of ascending elevation, include piñon-juniper woodlands (*Pinus edulis* Engelm., *Pinus monophylla* Torr. & Frem., *Juniperus osteosperma* (Torr.) Little, approx. 1370–2290 m), ponderosa pine woodlands and forests (*Pinus ponderosa* Lawson & C. Lawson, approx. 1950–2600 m), and mixed conifer forest (*Pinus ponderosa, Pseudotsuga menziesii* [Mirb.] Franco, *Picea engelmannii* Parry ex Engelm., *Abies lasiocarpa* [Hook.] Nutt., *Abies concolor* (Gord. & Glend.) Lindl. ex Hildebr., *Picea pungens* Engelm., *Populus tremuloides* Michx., approx. 2380–3000 m), with spruce-fir forests (*Picea engelmannii*, *Abies lasiocarpa*, approx. >2500 m) dominating the highest elevations (United States Department of the Interior (USDOI) National Park Service 2010). At lower elevations at the base of the plateau, annual precipitation averages 370 mm, summer maximum temperatures average 19.4 °C, and winter minimum temperatures average 4.5 °C; at higher elevations on the plateau, annual precipitation averages 710 mm, summer maximum temperatures average 14.3 °C, and winter minimum temperatures average − 0.3 °C (30-year normals for 1981–2010; https://prism.oregonstate.edu/normals/). Wildfire frequents the plateau (Fig. 1). In general, fires have historically been more frequent and lower in severity in ponderosa pine forest, and less frequent and of mixed severity in higher elevation forest types (Fulé et al. 2003a, b). Both planned and unplanned fire events are being used to restore ecosystem resilience and function to these fire-adapted ecosystems. Kaibab National Forest managers are actively restoring historic fire return intervals in ponderosa pine (Fire Regime I, 0–35 years) and mixed conifer (Fire Regimes III, IV, V, 35–200 years) forests of the Kaibab Plateau (USDA Forest Service 2014, 2020).

### Field observations

The USNPS and USFS maintain a field plot network to monitor and assess fire effects on vegetation and fuels within Grand Canyon National Park and the adjoining Kaibab National Forest (USDOI National Park Service 2010); field plot data are shared between the two federal agencies. Overstory trees are monitored on fixed-radius plots (area = 0.03 ha, radii = 10 m). For each tree with a diameter at breast height (DBH) > 15 cm, the following is periodically recorded: status (live or dead), DBH, species, height, live crown base height, and crown class (dominant, codominant, intermediate, subcanopy). Surface (downed woody, litter, and duff) fuels are monitored using one or two 15.24-m (50-ft) transects, with one end of the transect located at the center of the fixed-radius overstory plots. Both 1-h and 10-h fuels are measured along the first 1.83 m (6 ft) of transects, 100-h fuels are measured along the first 3.66 m (12 ft) of transects, and 1000-h fuels are measured along the entire length of the transects. Litter and duff depth are recorded at every 1.52 m (5 ft) along the length of transects. Transect starting point locations are recorded with professional-grade GNSS receivers and differentially corrected, resulting in expected horizontal accuracies of < 1 m. Overstory fixed-radius plot centers can be derived from these transect starting point locations.

We used a subset of overstory tree (*N* = 69) and surface fuel (*N* = 153) plot data that were spatially and temporally coincident (field observations taken within 2 years of airborne lidar acquisition) with 2012 and 2019 airborne lidar data for model development. Plots disturbed by fire between time of field measurement and lidar acquisition were not included. Available canopy fuel, defined as foliage weight plus 50% of small branch weight, was calculated allometrically for the overstory plots by implementing Appendix D of the FuelCalc User Guide in R (Reinhardt et al. 2006; Lutes 2021). Surface fuel transect tallies and depth measurements were converted to surface fuel density measurements following Brown et al. (1982) and averaged by plot.

### Airborne lidar data

Airborne lidar were acquired across 1853 and 2944 km^2^ of the Kaibab Plateau in 2012 and 2019, respectively (Fig. 1; Table 1). The 2012 lidar extent covered forested lands within the North Kaibab District of the Kaibab National Forest, as well as a portion of Grand Canyon National Park. The 2019 lidar extent spanned the entire North Kaibab District of the Kaibab National Forest. To measure fire-caused change and post-fire vegetation conditions, two smaller acquisitions totaling 175 km^2^ were made in 2020 across the entire 2019 Castle Fire extent and a portion of the 2019 Ikes Fire extent (42% coverage by 2019 and 2020 lidar). The Castle and Ikes Fires were 78 km^2^ and 67 km^2^ in size, respectively. Point cloud data, with points classified as ground or nonground, were delivered by vendors as tiled LAS files.

**Table 1:** Airborne lidar acquisition parameters for each acquisition

Parameter	Acquisition year		
	2012	2019	2020
Vendor	Watershed Sciences, Inc.	Atlantic	Technical Applications & Consulting, LLC
Platform	Cessna Caravan	PACVX (N750VX)	Cessna Turbo Utility 206
Sensor	Leica ALS50 Phase II and ALS60	Optech Galaxy Prime	Optech Galaxy T500
Acquisition dates	Aug. 25–Sept. 15, 2012	June 27–Jul. 3, 2019	Sept. 30–Oct. 2, 2020
Survey Altitude (AGL)	900–2000 m	1800 m	961–1066 m
Footprint diameter	21–45 cm	45 cm	24–27 cm
Scan frequency	49–66 Hz	53 Hz	110–113 Hz
Pulse rate of scanner	50–106 kHz	450 kHz	550 kHz
Laser wavelength	1064 nm	1064 nm	1064 nm
Mean pulse density	≥ 8 pulses m^− 2^	≥ 8 pulses m^− 2^	9–20 pulses m^− 2^
Total area surveyed	1853 km^2^	2944 km^2^	175 km^2^

Point cloud data were processed to create vegetation metrics with the LAStools (Isenburg 2021) and R (R Core Team 2021) software packages. Points were normalized to heights above ground with the “lasheight” LAStools function and vegetation metrics were calculated with “lascanopy” LAStools function (Table 2). Metrics were calculated for circular fixed-radius (radii = 10 m) overstory plot extents coincident with 2012 and 2019 lidar data (*N *= 69), and for circular areas encompassing 15.24-m surface fuel transects (*N* = 153), to be used for predictive modeling. Metric grids with a spatial resolution of 20 m were also created by binning lidar points into 20-m grid cells (step = 20) across lidar extents with “lascanopy.” These grids were used for mapping. Topographic metrics based on vendor-supplied digital terrain models (DTM) were created at 20-m spatial resolution with the “raster” and “spatialEco” R packages (Table 2; Hijmans 2021a; Evans 2021). Topographic metric values at plot locations were extracted for use in predictive modeling.

**Table 2:** Vegetation and topography metrics, derived from airborne lidar, and fire history metrics, derived from the Monitoring Trends in Burn Severity (MTBS) database. Metrics were candidate predictor variables for random forest (RF) models predicting fuel loads. Nonground returns are defined as those > 0 m above ground. Canopy returns are defined as those > 2 m above ground. Understory returns are defined as those > 0 and < 2 m above ground

Metric name	Description
MAX.gt2	Maximum height of canopy returns
AVG.gt2	Mean height of canopy returns
STD.gt2	Standard deviation of canopy return heights
SKE.gt2	Skewness of canopy return heights
KUR.gt2	Kurtosis of canopy return heights
P05.gt2	5th percentile of canopy return heights
P10.gt2	10th percentile of canopy return heights
P25.gt2	25th percentile of canopy return heights
P50.gt2	50th percentile of canopy return heights
P75.gt2	75th percentile of canopy return heights
P90.gt2	90th percentile of canopy return heights
P95.gt2	95th percentile of canopy return heights
MAX.lt2	Maximum height of understory returns
AVG.lt2	Mean height of understory returns
STD.lt2	Standard deviation of understory return heights
SKE.lt2	Skewness of understory return heights
KUR.lt2	Kurtosis of understory return heights
P05.lt2	5th percentile of understory return heights
P10.lt2	10th percentile of understory return heights
P25.lt2	25th percentile of understory return heights
P50.lt2	50th percentile of understory return heights
P75.lt2	75th percentile of understory return heights
P90.lt2	90th percentile of understory return heights
P95.lt2	95th percentile of understory return heights
D00	Percentage of nonground returns 0–0.5 m above ground
D01	Percentage of nonground returns 0.5–1 m above ground
D02	Percentage of nonground returns 1–2 m above ground
D03	Percentage of nonground returns 2–4 m above ground
D04	Percentage of nonground returns 4–8 m above ground
D05	Percentage of nonground returns 8–16 m above ground
D06	Percentage of nonground returns 16–32 m above ground
D07	Percentage of nonground returns 32–48 m above ground
D00.lt2	Percentage of understory returns 0–0.05 m above ground
D01.lt2	Percentage of understory returns 0.05–0.15 m above ground
D02.lt2	Percentage of understory returns 0.15–0.5 m above ground
D03.lt2	Percentage of understory returns 0.5–1 m above ground
D04.lt2	Percentage of understory returns 1–2 m above ground
CURV.plan	Planform curvature (Zevenbergen and Thorne 1987)
CURV.profile	Profile curvature (Zevenbergen and Thorne 1987)
CURV.total	Total curvature (Zevenbergen and Thorne 1987)
DEM	Elevation
HLI	Heatload index (Eq. 3 of McCune and Keon 2002)
SLOPE	Slope (degrees)
SCOSA	Slope * cosine(aspect) (Stage 1976)
SSINA	Slope * sin(aspect) (Stage 1976)
TPI	Topographic position index (Hijmans 2021a)
TRASP	Transformed aspect (1-cosine(aspect – 30))/2) (Roberts and Cooper 1989)
TRI	Topographic roughness index (Wilson et al. 2007)
YSF	Years since fire (derived from the MTBS database, 1984–2019)
NPF	Number of past fires (derived from the MTBS database, 1984–2019)

### Landsat fire history data

We used the Monitoring Trends in Burn Severity (MTBS) database (1984–2019; Eidenshink et al. 2007) coincident with the study area (Fig. 1) to create years-since-fire (YSF) and number-of-past-fire (NPF) grids for the years 2012 and 2019. Grids were created by converting MTBS polygons representing fire perimeters to 20-m raster format and performing raster calculations with the “terra” package in R (Hijmans et al. 2021b). YSF and NPF values were extracted at plot locations to be used as additional predictor variables, and YSF and NPF grids were used for mapping.

### Random forest modeling

We predicted canopy and surface fuels from airborne lidar and Landsat-derived fire history metrics using random forest (RF) modeling implemented in the “randomForest” package of R (Breiman 2001; Liaw and Wiener, 2002; R Core Team 2021). Response variables included available canopy fuel, 1- to 1000-h fuels, litter and duff, and total surface fuel (sum of 1- to 1000-h, litter and duff fuels). Candidate predictor variables included the airborne lidar and fire history metrics listed in Table 2.

For each response variable, we identified the most important predictor variables using the “rf.modelSel” routine of the “rfUtilities” R package (Evans and Murphy 2018; Murphy et al. 2010), which computes normalized predictor variable importance scores (MIR) that range from 1 (most important) to zero (least important). To reduce possible bias towards selection of highly correlated predictor variables (Strobl et al. 2008), we considered only one predictor variable of highly correlated predictor variable pairs or sets (*r *> 0.9) when running the “rf.modelSel” routine, which we ran with various predictor variable sets. RF models were run in regression mode with the default values of 500 trees (ntree = 500) and the number of variables at each node split set to the total number of candidate predictor variables (p) divided by three (mtry = p/3). Model performance was assessed with out-of-bag error estimates. Final RF models for each of the four response variables included only the most important, not highly correlated, predictor variables.

### Fuel map creation and analysis

Final RF models predicting available canopy fuel and total surface fuel were applied to 20-m metric grids to create maps of these two fuel variables across each lidar acquisition (years 2012, 2019, 2020). We analyzed 2012 and 2019 predicted fuel maps by time since fire, as recorded by MTBS-derived YSF grids, and forest type, as mapped by LANDFIRE Existing Vegetation Type (EVT) grids (LANDFIRE 2014, 2016). We explored linear and various nonlinear models for describing relationships between predicted fuel maps and YSF.

Consumption was estimated across the 2019 Castle Fire extent and a portion of the 2019 Ikes Fire extent by differencing 2019 and 2020 predicted fuel maps. To test whether consumption was related to burn severity, we compared consumption grids with MTBS differenced Normalized Burn Ratio (dNBR) grids, indicators of burn severity (Key and Benson 2006). Total and average consumption for the Castle Fire and portion of the Ikes Fire were estimated by summing and averaging mapped consumption estimates within fire extents. Confidence intervals for total consumption were created by multiplying total consumption estimates by percent root mean square error (%RMSE) values from RF models. %RMSE was defined as the square root of the mean of the squared residuals, divided by the mean of the observed fuel values. Grid cells within the 2018 Stina Fire extent were excluded from the calculation of consumption averages and totals for the Ikes Fire.

## Results

### Random forest models predicting fuel loads

**Fig. 2:**
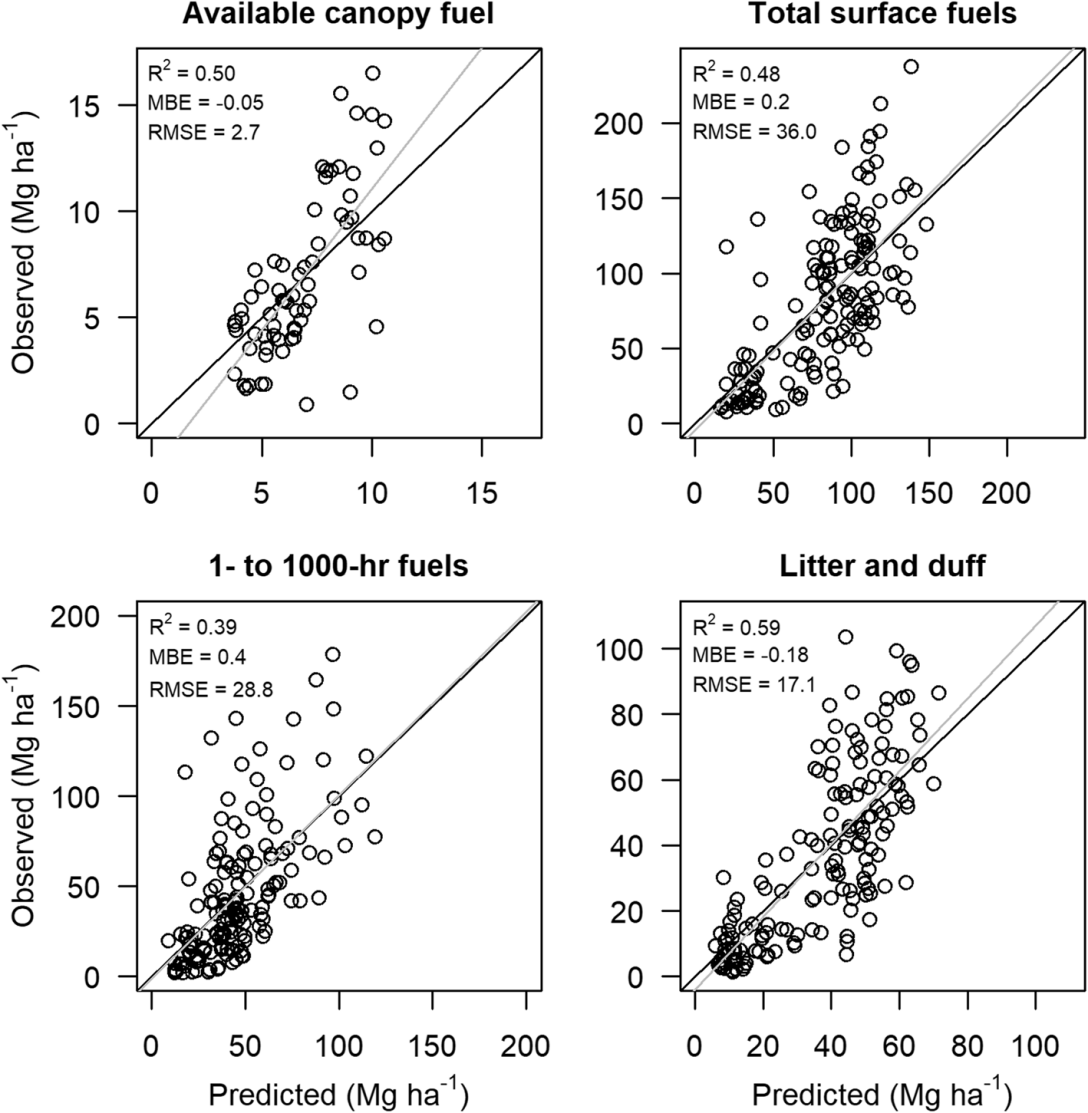
Observed versus predicted available canopy (*N* = 69) and surface (*N* = 153) fuel loads. Fuel loads were predicted from airborne lidar and fire history metrics at field plot locations with random forest (RF) models. Mean bias error (MBE) is the mean of the predicted values minus the mean of the observed values. Root mean square error (RMSE) is the square root of the mean of the squared residuals, where residuals are observed minus predicted values. 1:1 lines are shown in black, and fit lines are shown in gray. MBE and RMSE are in units of Mg ha^− 1^

Available canopy fuel ranged from 0.9 to 16.5 Mg ha^− 1^ and averaged 6.9 Mg ha^− 1^ across the 69 fixed-radius plots. Our RF model explained 50% of the variation in available canopy fuel (Fig. 2). Variables describing canopy height distribution (SKE.gt2, KUR.gt2, P10.gt2, P50.gt2) and density of lower vegetation (D00, D03, D02.lt2) as well as one topographic interaction variable between slope and aspect (SSINA) were important predictors of available canopy fuel (Table 3).

**Table 3:** Important predictors included in final models and percent variance explained for each fuel response variable. Predictor variables are ordered by importance, and normalized predictor variable importance scores (MIR) ranging from 1 (most important) to zero (least important) are given in parentheses after each predictor variable. See Table 2 for predictor variable definitions

Response variable	Important predictors	Var. Exp. (%)
Available canopy fuel	P10.gt2 (1), SKE.gt2 (0.6), D03 (0.6), P50.gt2 (0.5), KUR.gt2 (0.4), D00 (0.4), D02.lt2 (0.3), SSINA (0.3)	50
1- to 1000-h fuels	DEM (1), P05.gt2 (1), D01 (0.6), D00.lt2 (0.6), NPF (0.3)	39
Litter and duff	DEM (1), YSF (0.6), NPF (0.4), D06 (0.3), D03 (0.3), D00 (0.2),	59
Total surface fuel	DEM (1), D03.lt2 (0.6), D02.lt2 (0.5), P90.lt2 (0.5), P10.gt2 (0.5), YSF (0.3), NPF (0.2)	48

Across the 153 fuel transects, 1- to 1000-h fuels ranged from 2.1 to 178.6 Mg ha^− 1^ and averaged 45.8 Mg ha^− 1^, duff and litter ranged from 1.4 to 103.6 Mg ha^− 1^ and averaged 36.9 Mg ha^− 1^, and total surface fuels ranged from 7.7 to 237.6 Mg ha^− 2^ and averaged 82.7 Mg ha^− 1^. RF models explained 39% of the variation in 1- to 1000-h fuels, 59% of the variation in litter and duff, and 48% of the variation in total surface fuels (Fig. 2). Lower canopy height (P05.gt2), understory density (D01, D00.lt2), elevation (DEM), and number of past fires (NPF) were important predictors of 1- to 1000-h fuels that were included in the final RF model (Table 3). Understory (D00) and canopy (D03, D06) density, elevation (DEM), and fire history (YSF, NPF) variables were important predictors of litter and duff (Table 3). Important predictors of total surface fuels included in the final model were lower canopy height (P10.gt2), understory height (P90.lt2), understory density (D02.lt2, D03.lt2), elevation (DEM), and fire history (YSF, NPF) variables (Table 3).

### Fuel and consumption map analyses

**Fig. 3:**
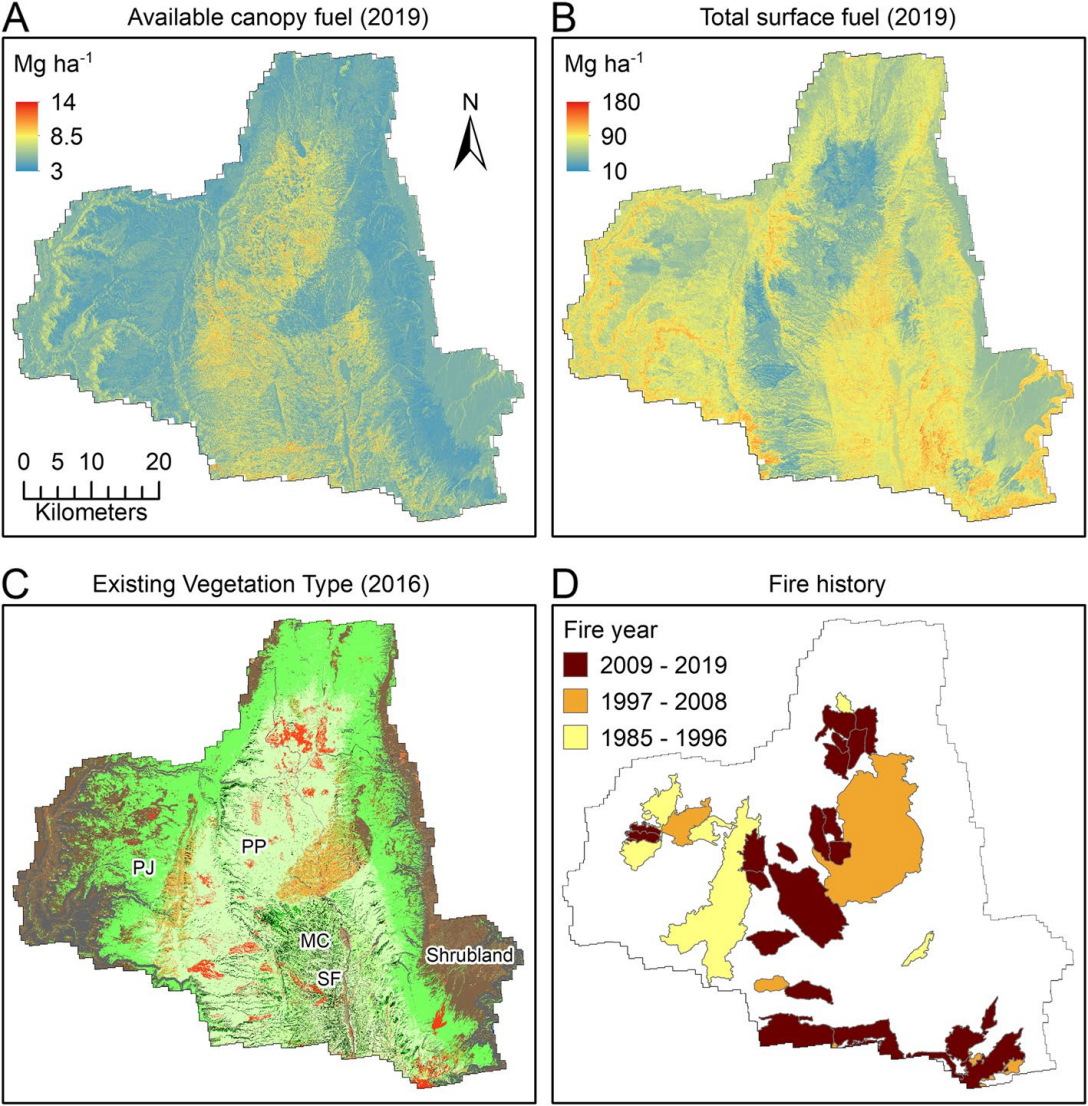
Maps of predicted available canopy fuel in 2019 (**A**), predicted total surface fuel in 2019 (**B**), LANDFIRE Existing Vegetation Types in 2016 (**C**), and MTBS fire perimeter polygons from 1984 to 2019 (**D**). Existing Vegetation Type labels: piñon-juniper (PJ), ponderosa pine (PP), mixed-conifer (MC), and spruce-fir (SF)

Predicted available canopy fuel maps varied with LANDFIRE EVT as expected, with greater available canopy fuels in ponderosa pine, mixed-conifer, and spruce-fir forests on the Kaibab Plateau, and less available canopy fuels in piñon-juniper woodlands and shrublands at lower elevations (Fig. 3). There was no significant relationship between available canopy fuel and years since fire.

**Fig. 4:**
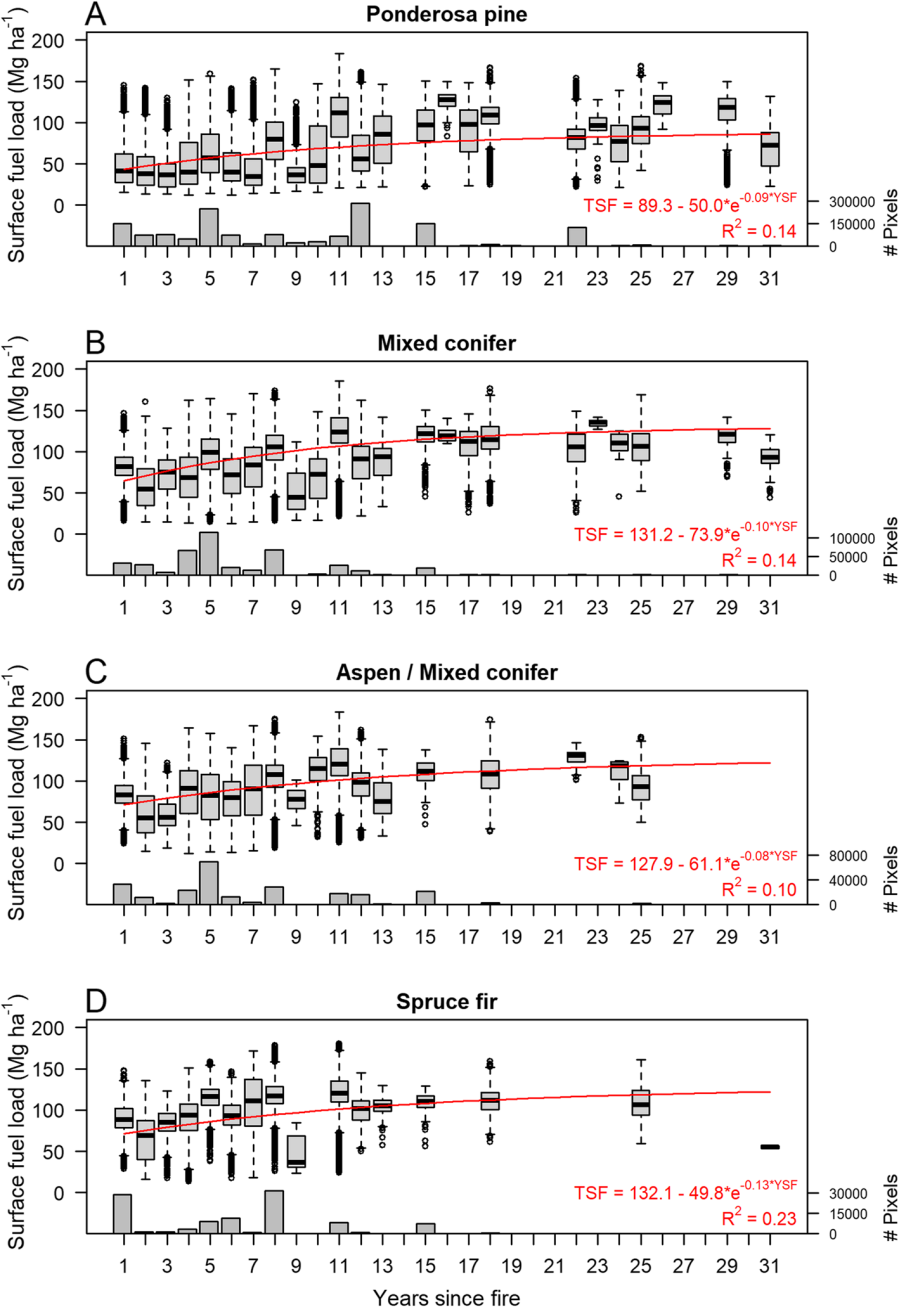
Time series of surface fuel loads derived from overlaying 2012 and 2019 predicted surface fuel load (TSF) maps with years since fire (YSF) grids created from the Monitoring Trends in Burn Severity (MTBS) database (1984–2019). Predicted surface fuel loads increased with years since fire, with asymptotes where fuel accumulation slowed at approximately 10–15 years post fire. Fit lines for three-parameter asymptotic models are shown as red lines. The number of 20-m pixels that went into each distribution is quantified in bars at the bottom of each panel

Predicted total surface fuel maps showed variation related to LANDFIRE EVT and fire history; ponderosa pine forests and recently burned areas tended to have lower total surface fuel loads (Fig. 3). Total surface fuel loads were positively and significantly related to years since fire (Fig. 4). Three-parameter asymptotic models described the relationship between predicted total surface fuels and years since fire slightly better than linear models, with *R*^2^ values ranging from 0.10 to 0.23. The response of surface fuel to time since fire followed an asymptote towards stable fuel levels of approximately 90–130 Mg ha^− 1^ at 10–15 years post-fire (Fig. 4). More observations (20-m pixels) were available in areas that burned between 1 and 15 years previously, with relatively fewer observations available for areas that burned > 15 years previously.

**Fig. 5:**
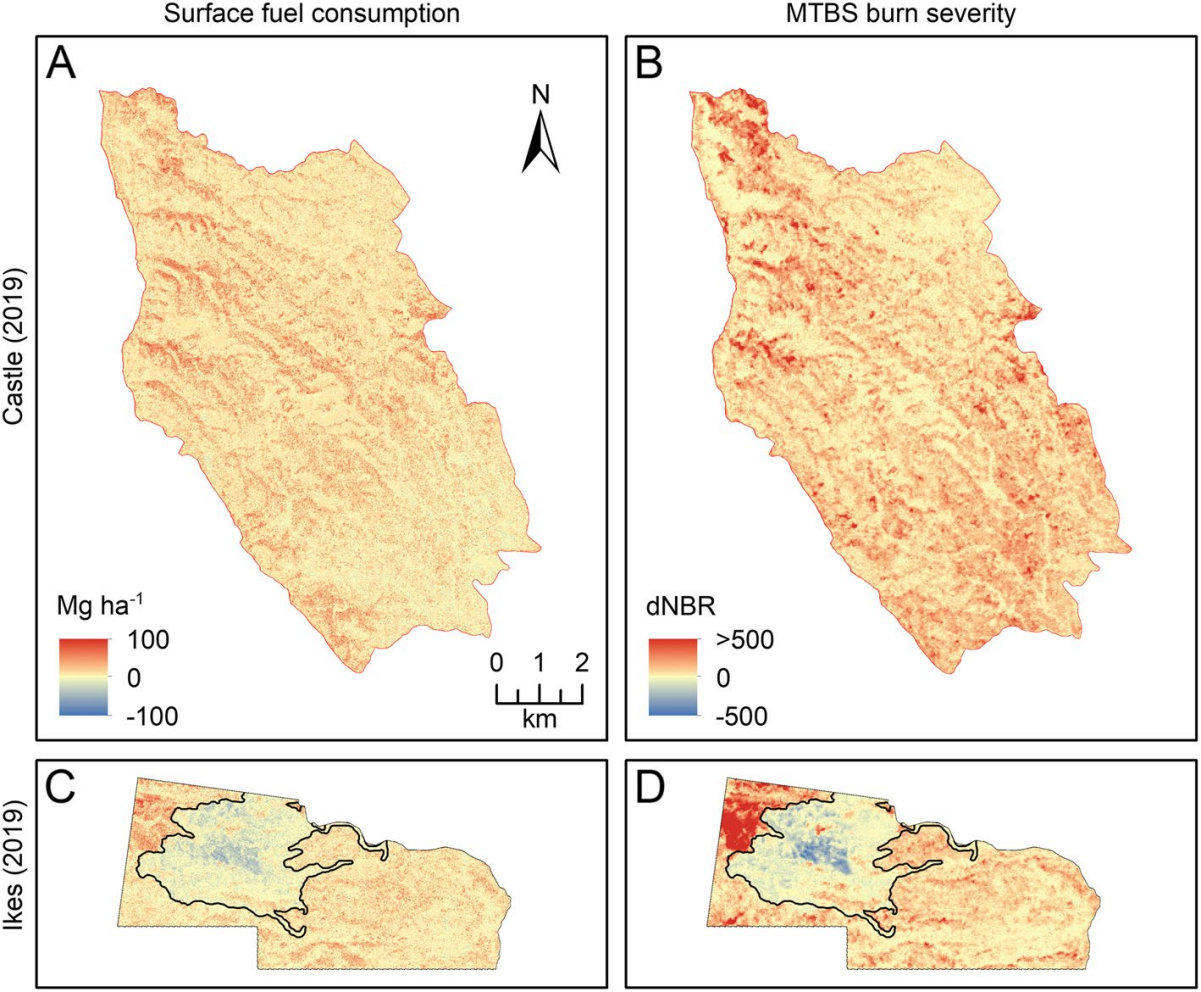
Maps of predicted surface fuel consumption and MTBS burn severity, as indicated by the differenced Normalized Burn Ratio (dNBR), for the 2019 Castle Fire and a portion of 2019 Ikes Fire. Surface fuel consumption was positively correlated with burn severity. The 2018 Stina Fire perimeter within the 2019 Ikes Fire is represented by a black line

Predicted surface fuel consumption averaged 16.1 and 14.0 Mg ha^− 1^ for the Castle and Ikes Fires, respectively (Fig. 5). Total surface fuel consumed by the Castle Fire and the portion of the Ikes Fire where pre- and post-fire lidar data were available was estimated to be 125.3 ± 54.6 and 27.6 ± 12.0 Gg, respectively. Predicted canopy fuel consumption averaged − 0.3 Mg ha^− 1^ across both fires, i.e., on average, available canopy fuel increased between 2019 and 2020. For areas that burned at high severity, predicted canopy fuel consumption averaged 0 and 0.09 Mg ha^− 1^ for the Castle Fire and portion of the Ikes Fire, respectively. Predicted canopy fuel consumption for the portion of the Ikes Fire totaled 0.003 ± 0.001 Gg. The 2018 Stina Fire extent (not included in consumption averages and totals) was apparent in the 2019 Ikes Fire extent as an area of “negative” consumption (Fig. 5).

Surface fuel consumption grids were positively correlated with the dNBR grids, with Pearson correlation coefficients of 0.33 and 0.37 for the Castle and Ikes Fires, respectively (Fig. 5). Average predicted surface fuel consumption varied by MTBS burn severity class, with smallest average consumption in the unburned-to-low severity class and greatest average consumption in the high severity class (Table 4). Canopy fuel consumption and dNBR grids were uncorrelated, with Pearson correlation coefficients of 0 and 0.04 for the Castle and Ikes Fires, respectively.

**Table 4:** Average and standard deviation (in parentheses) of predicted surface fuel consumption, in Mg ha^− 1^, by MTBS burn severity class for the 2019 Castle and Ikes Fires

MTBS burn severity class	Fire	
	Castle	Ikes
Unburned to low	10.7 (11.3)	8.7 (12.0)
Low	17.5 (15.8)	15.6 (15.6)
Moderate	29.6 (19.3)	29.8 (20.3)
High	41.5 (21.4)	30.1 (19.0)

## Discussion

Our results show an encouraging improvement in estimating and mapping fuel loads and consumption with airborne lidar, especially for subcanopy fuels that have been poorly characterized with remote sensing in the past. We predicted canopy and surface fuel loads with moderate accuracy (39–59%) including both airborne lidar and fire history predictor variables. Our analysis focused on surface fuel loads because fewer previous studies have predicted surface fuels with airborne lidar, and because canopy fuel available for burning was much smaller relative to surface fuel in our study area. Our moderate accuracies for surface fuel load prediction with airborne lidar are comparable to those of past studies (Table 5).

**Table 5:** Other studies predicting surface fuel variables from airborne lidar

Authors	Year	Forest type	Location	Surface fuel response variable(s)	Variance explained (%)
Pesonen et al.	2008	Spruce and hardwood	Finland	Downed dead wood volume	61
Jakubowski et al.	2013	Mixed conifer	California, USA	1000-h fuel load, fuel bed depth	31, 35
Hudak et al.	2015	Longleaf pine savanna	Florida, USA	ln(surface fuel load)	44
Price and Gordon	2016	Dry Sclerophyll Forest	Australia	Surface fuel load	24
Bright et al.	2017	Pine and spruce-fir	Colorado, USA	Litter and duff, 1- to 100-h, 1000-h, and total surface fuel loads	24–32
Stefanidou et al.	2020	Fir	Greece	Transformed litter, grass/forbs, 1-h, 10-h, and total surface fuel loads	60–71
McCarley et al.	2020	Conifer	New Mexico and Oregon, USA	Understory fuel load	16–63
Mauro et al.	2020	Conifer	Oregon, USA	Downed woody biomass	14
Alonso-Rego et al.	2021	Pine	Spain	Understory fuel, litter and duff, and downed woody debris loads	35–42
Bright et al. (this study)	2022	Pine, mixed-conifer, spruce-fir	Arizona, USA	Litter and duff, 1- to 1000-h and total surface fuel loads	39–59

Our RF models showed relatively high predictive power for low fuel loads but showed a trend of increased error and underprediction for higher fuel loads (Fig. 2). Although nonparametric RF models do not require normally distributed variables, underprediction at the high end of fuel gradients might have been caused by field data being skewed toward the lower end of fuel gradients. Most field data were collected at 10 years or less post-fire, for the intent of monitoring post-fire recovery. Including more field sampling at the higher end of fuel gradients in areas that had not recently burned might have helped to alleviate this increased error and under prediction of higher fuel loads. We were, however, limited to using field data that were not gathered for the intent of our modeling analysis, nor did we try to balance samples by excluding any of the limited number of available field observations.

RF models predicting surface fuel loads included both near surface (0–2 m aboveground) and overstory (> 2 m above ground) predictor variables, indicating that airborne lidar both directly measured variation in near surface fuels and indirectly captured surface fuel variation through overstory correlates. Our RF model predicting litter and duff, which cannot be directly measured with airborne lidar as it does not penetrate the ground surface, especially demonstrates the potential of using overstory correlates to estimate underlying surface fuels. Others have also found that overstory correlates are helpful for predicting surface fuel variables with airborne lidar (Price and Gordon 2016; Bright et al. 2017; Stefanidou et al. 2020; McCarley et al. 2020) and have documented relationships between overstory characteristics and surface fuel loads (Prescott 2002; Lydersen et al. 2015; López-Senespleda et al. 2021).

One topographic variable, elevation, was an especially important predictor of surface fuel loads across our study area on the Kaibab Plateau in Arizona. Surface fuel increased with elevation, and we predicted smaller surface fuel loads in ponderosa pine forests relative to higher elevation forest types. This pattern is likely because of increasing annual precipitation with elevation that results in greater vegetation growth, ecosystem productivity, and therefore fuel loads. Environmental gradients such as elevation, when combined with other data sources, have proven to be useful in other fuel mapping efforts (Keane et al. 2000, 2001; Reich et al. 2004; Pierce et al. 2012; Lin et al. 2021).

Predicted surface fuel loads varied significantly with time since fire, as measured by MTBS Landsat products, across our study area (Fig. 4). Although Landsat-derived fire history variables were not as important as lidar variables describing vegetation or elevation, they helped explain variation in surface fuels unexplained by lidar variables. Mapped total surface fuel loads increased with time since fire until about 10–15 years post fire, after which predicted fuel loads approached a steady state. Relatively fewer pixels had burned between 15 and 31 years previously in our study area (Fig. 4) so that the relationship between surface fuel load and time since fire that we reported is less reliable for that time period. Nevertheless, our asymptotic models are conceptually close to classic fire-driven fuel accumulation models such as Olson’s negative exponential equation (Olson 1963; Birk and Simpson 1980; Keane 2015; Zazali et al. 2020). Likewise, previous field observation-based studies in ponderosa pine forests (Roccaforte et al. 2012) and mixed conifer forests (Dunn and Bailey 2015; Eskelson and Monleon, 2018; Stevens-Rumann et al. 2020) have documented similar asymptotic temporal trends in post-fire surface fuel loads that reached a steady state at 6–20 years post fire. Fine fuel accumulation is the balance between the input and the removal of fuels, mainly driven by litterfall and decomposition (Hanan et al. 2022). Litter accumulates on the soil until litterfall equals decomposition and accumulation stabilizes around a mean steady state (Ewel et al. 1976). Note as well that both decomposition and litterfall are complex processes mainly driven by climate, aboveground biomass, site, and soil conditions (Prescott 2002; Bezkorovaynaya 2005; Krishna and Mohan 2017; Neumann et al. 2018; Costa et al. 2020). Changes to these can alter the system feedbacks and affect the accumulation process which would explain the fluctuation of fuel loads over the asymptote after a long time since fire (Fig. 4). In our study area, regions of lower productivity and therefore infrequent burning might also be responsible for seemingly stable fuel loads > 15 years post fire. To our knowledge, few studies have quantified the relationship between remote sensing-estimated fuel loads and time since fire. In longleaf pine forest in Florida, Hudak et al. (2016) also documented meaningful correlations between fuel loads estimated from airborne lidar and time since fire.

Differencing pre- and post-fire fuel load maps allowed us to estimate fuel consumption across fire extents. Both fires were dominated by low severity fire as indicated by MTBS dNBR (90–96%), which generally corresponds to non-crown fire in this area (Hoff et al. 2019); therefore, on average across fire extents, available canopy fuel was greater in 2020 than it was in 2019. Our maps did, however, document available canopy fuel consumption in areas that burned severely, as indicated by dNBR. Low canopy fuel consumption (average of 0.07 Mg ha^− 1^ for pixels that burned severely) relative to surface fuel consumption (average of 14.0 and 16.1 Mg ha^− 1^) was expected, as surface fuel loads were an order of magnitude greater. dNBR was moderately correlated with surface fuel consumption, but uncorrelated with canopy fuel consumption, likely because little to no canopy fuel burned in these fires which burned predominantly at low severity. For other fires where crown fire is more prevalent, dNBR would likely be related more strongly to canopy fuel consumption. Our finding of moderate correlation between dNBR and surface fuel consumption suggests that dNBR could possibly be used as an index of surface fuel consumption for low-severity fires, although physically based estimates of consumption derived from pre- and post-fire lidar are likely superior. Our estimates of average fuel consumption (14.0–16.1 Mg ha^− 1^) and total surface fuel consumption (125.3 ± 54.6 and 27.6 ± 12.0 Gg for the Castle and portion of the Ikes Fires, respectively) are similar in magnitude but less than those of McCarley et al. (2020), who estimated average total fuel consumptions of 45–66 Mg ha^− 1^ and consumption totals ranging from 224 to 713 Gg for a portion of the 2011 Las Conchas Fire (49 km^2^) in New Mexico and the 2012 Pole Creek Fire (108 km^2^) in Oregon. As multitemporal lidar becomes more common, additional similar analyses estimating consumption will be possible. Such consumption estimates can increase our understanding of land/atmosphere exchanges of carbon.

## Conclusions

Airborne lidar, when combined with field observations, can be used to predict and map canopy and surface fuel loads with moderate accuracy across landscapes. We found that surface fuel loads were related to time since fire and present a remote sensing framework for quantifying landscape temporal dynamics in surface fuel accumulation. Our finding that fuel loads were related to time since fire suggests that future work that aims to map fuel loads with remote sensing can benefit from considering fire and other disturbance history.

Landscape scale fuel load maps derived from active remote sensing can provide unbiased geospatial decision support information to forest, wildland fire, and wildlife managers, helping them assess current conditions and plan future treatments for wildland fire risk reduction and long-term ecosystem resilience. As airborne lidar becomes more common in forested landscapes, our methods can also serve as a framework for estimating landscape scale fire emissions and assessing ecosystem dependent relationships, such as post disturbance fuel loading trajectories. The novel approaches described here can be especially useful in landscapes prone to uncharacteristically high severity wildfire due to climate change, such as the sky islands of the southwestern United States.

## Data Availability

The datasets used and analyzed during the current study are available from the corresponding author on reasonable request. Fuel load and consumption maps are available on WIFIRE Commons at 10.48792/W2MW2V.

## Abbreviations

DBH: Diameter at breast height
dNBR: Differenced Normalized Burn Ratio
DTM: Digital terrain model
EVT: Existing Vegetation Type
Lidar: Light detection and ranging
MBE: Mean bias error
MIR: Normalized predictor variable importance score
MTBS: Monitoring Trends in Burn Severity
NPF: Number of past fires
RF: Random forest
RMSE: Root mean square error
%RMSE: Percent root mean square error
USFS: United States Forest Service
USNPS: United States National Park Service
YSF: Years since fire
